# Comparative Genomics Guides Elucidation of Vitamin B_12_ Biosynthesis in Novel Human-Associated *Akkermansia* Strains

**DOI:** 10.1128/AEM.02117-19

**Published:** 2020-01-21

**Authors:** Nina Kirmiz, Kadir Galindo, Karissa L. Cross, Estefani Luna, Nicholas Rhoades, Mircea Podar, Gilberto E. Flores

**Affiliations:** a Department of Biology, California State University, Northridge, Northridge, California, USA; b Biosciences Division, Oak Ridge National Laboratory, Oak Ridge, Tennessee, USA; c Microbiology Department, University of Tennessee, Knoxville Tennessee, USA; d Department of Molecular Biology and Biochemistry, University of California, Irvine, Irvine, California, USA; Chinese Academy of Sciences

**Keywords:** *Akkermansia*, human gut microbiome, intestinal bacteria, probiotics, vitamin B_12_

## Abstract

Akkermansia muciniphila is a mucin-degrading bacterium found in the gut of most humans and is considered a “next-generation probiotic.” However, knowledge of the genomic and physiological diversity of human-associated *Akkermansia* sp. strains is limited. Here, we reconstructed 35 metagenome-assembled genomes and combined them with 40 publicly available genomes for comparative genomic analysis. We identified at least four species-level phylogroups (AmI to AmIV), with distinct functional potentials. Most notably, we identified genes for cobalamin (vitamin B_12_) biosynthesis within the AmII and AmIII phylogroups. To verify these predictions, 10 *Akkermansia* strains were isolated from adults and screened for vitamin B_12_ biosynthesis genes via PCR. Two AmII strains were positive for the presence of cobalamin biosynthesis genes, while all 9 AmI strains tested were negative. To demonstrate vitamin B_12_ biosynthesis, we measured the production of acetate, succinate, and propionate in the presence and absence of vitamin supplementation in representative strains of the AmI and AmII phylogroups, since cobalamin is an essential cofactor in propionate metabolism. Results showed that the AmII strain produced acetate and propionate in the absence of supplementation, which is indicative of vitamin B_12_ biosynthesis. In contrast, acetate and succinate were the main fermentation products for the AmI strains when vitamin B_12_ was not supplied in the culture medium. Lastly, two bioassays were used to confirm vitamin B_12_ production by the AmII phylogroup. This novel physiological trait of human-associated *Akkermansia* strains may affect how these bacteria interact with the human host and other members of the human gut microbiome.

**IMPORTANCE** There is significant interest in the therapeutic and probiotic potential of the common gut bacterium Akkermansia muciniphila. However, knowledge of both the genomic and physiological diversity of this bacterial lineage is limited. Using a combination of genomic, molecular biological, and traditional microbiological approaches, we identified at least four species-level phylogroups with differing functional potentials that affect how these bacteria interact with both their human host and other members of the human gut microbiome. Specifically, we identified and isolated *Akkermansia* strains that were able to synthesize vitamin B_12_. The ability to synthesize this important cofactor broadens the physiological capabilities of human-associated *Akkermansia* strains, fundamentally altering our understanding of how this important bacterial lineage may affect human health.

## Abstract (precis)

There is significant interest in the therapeutic and probiotic potential of the common gut bacterium Akkermansia muciniphila. However, knowledge of both the genomic and physiological diversity of this bacterial lineage is limited. Using a combination of genomic, molecular biological, and traditional microbiological approaches, we identified at least four species-level phylogroups with differing functional potentials that affect how these bacteria interact with both their human host and other members of the human gut microbiome. Specifically, we identified and isolated *Akkermansia* strains that were able to synthesize vitamin B_12_. The ability to synthesize this important cofactor broadens the physiological capabilities of human-associated *Akkermansia* strains, fundamentally altering our understanding of how this important bacterial lineage may affect human health.

## INTRODUCTION

Akkermansia muciniphila is a mucin-degrading, Gram-negative, intestinal bacterium that is widely present in the human population, typically at 1% to 4% relative abundance (1, 2). A number of studies in humans (3–5) and rodents (6–8) have found positive associations between its abundance and intestinal health, suggesting that *Akkermansia* might be a beneficial member of the gut microbiome and could be used as a biomarker of a healthy gut (9–11). However, despite a diversity of phylotypes being reported in previous sequence-based studies, A. muciniphila Muc^T^ (ATCC BAA-835) represents the only described species of the *Verrucomicrobia* phylum associated with humans (2, 12, 13). Therefore, before we can fully assess the health potential of human-associated *Akkermansia* strains, a comprehensive understanding of the genomic and physiological diversity of this lineage is needed.

Recently, a pangenomic study that included 33 new isolates from adults and 6 from laboratory mice provided insights into the population structure and evolutionary history of the *Akkermansia* lineage (14). Specifically, this study revealed an open pangenome with at least three species-level phylogroups (AmI, AmII, and AmIII), which appear to be evolving independently. Although genomic differences among phylogroups were noted, the physiological consequences were not explored.

To continue to expand our understanding of the genomic content and functional potential of human-associated *Akkermansia* strains, we reconstructed 35 *Akkermansia* genomes from children 2 to 9 years of age and combined our genomes with those reported by Guo et al. (14). With these genomes, we identified novel diversity and several putative functional differences among the *Akkermansia* phylogroups. Most notably, we identified the presence of genes associated with *de novo* cobalamin (vitamin B_12_) biosynthesis in selected phylogroups of *Akkermansia*. Furthermore, using isolates obtained from healthy adults, we tested these genomic predictions and confirmed vitamin B_12_ biosynthesis by select human-associated *Akkermansia* strains. These results build on our understanding of the physiological capabilities of human-associated *Akkermansia* strains and demonstrate an important biosynthetic activity for this bacterial lineage, which further expands its potential beneficial role in the intestinal environment.

## RESULTS

### Comparative genomics.

A total of 334.9 Gbp of metagenomic sequence data were obtained from 70 children 2 to 9 years of age. Using SPAdes (15) to assemble contigs and MetaBAT (16) to bin contigs, we recovered 35 high-quality metagenome-assembled genomes (MAGs) of human-associated *Akkermansia* strains from 35 of the 70 children (Table 1). The completeness of the MAGs was relatively high, ranging from 68.5% to 95.5%, with 31 of 35 MAGs being >90% complete. Similarly, contamination of the MAGs was low (<1% for all). On average, each MAG was 2.87 Mbp in length and contained approximately 2,420 protein-coding genes.

**TABLE 1:** Summary of 35 *Akkermansia* MAGs recovered from a diverse population of children 2 to 9 years of age, living in Los Angeles, California

Genome name	Genome properties					Assembly properties		
	Phylogroup	% completeness	% contamination	No. of predicted proteins	% coding density	Contigs	Length (Mbp)	% GC content
A. muciniphila Muc^T^	AmI	100	0.0	2,238	88.6	1	2.66	55.8
CDI-75C-7	AmI	95.5	0.0	2,433	88.4	26	2.82	55.5
CDI-92A-19	AmI	68.5	0.0	2,117	89.0	317	2.27	55.8
CDI-93C-15	AmI	91.0	0.0	2,345	88.8	231	2.59	55.8
CDI-16B-22	AmI	94.6	0.0	2,291	88.5	79	2.65	55.6
CDI-158B-12	AmI	95.5	0.0	2,302	88.2	42	2.70	55.3
CDI-50B-13	AmI	95.5	0.0	2,293	88.4	22	2.72	55.6
CDI-51A-11	AmI	95.5	0.0	2,295	88.4	22	2.72	55.6
CDI-28A-8	AmI	95.5	0.0	2,301	88.2	27	2.71	55.4
CDI-85A-12	AmI	95.5	0.0	2,225	88.4	19	2.66	55.4
CDI-30A-11	AmI	95.5	0.0	2,272	88.4	22	2.68	55.5
CDI-42C-15	AmI	95.5	0.0	2,340	88.4	25	2.74	55.2
CDI-151B-10	AmI	94.6	0.0	2,383	88.3	64	2.77	55.4
CDI-193A-6	AmI	95.5	0.0	2,416	88.3	32	2.83	55.4
CDI-143C-7	AmII	81.1	0.9	2,301	88.4	206	2.67	58.7
CDI-10B-12	AmII	94.6	0.0	2,439	88.1	32	2.98	58.3
CDI-128B-11	AmII	92.8	0.0	2,428	88.1	22	2.96	58.3
CDI-129B-12	AmII	88.3	0.0	2,375	88.1	229	2.70	58.5
CDI-77C-9	AmII	95.5	0.0	2,435	88.0	25	2.99	58.2
CDI-24B-9	AmII	94.6	0.0	2,450	88.2	31	3.02	58.1
CDI-182B-6	AmII	95.5	0.0	2,478	88.1	22	3.02	58.3
CDI-198C-9	AmII	95.5	0.0	2,512	88.3	51	3.00	58.2
CDI-69C-9	AmII	95.5	0.0	2,421	88.2	24	2.96	58.2
CDI-138A-11	AmII	95.5	0.0	2,483	88.0	25	3.01	58.2
CDI-70C-8	AmII	95.5	0.0	2,481	88.0	32	3.01	58.2
CDI-26A-8	AmII	92.8	0.9	2,610	88.1	251	2.95	58.0
CDI-34A-8	AmII	95.5	0.0	2,558	87.2	29	3.09	57.8
CDI-65B-6	AmII	87.4	0.0	2,538	87.4	55	3.04	57.8
CDI-203B-7	AmII	94.6	0.9	2,479	87.8	25	2.99	58.1
CDI-150B-9	AmIV	95.5	0.0	2,457	87.7	29	2.99	57.2
CDI-12C-16	AmIV	95.5	0.0	2,461	87.8	32	2.99	57.2
CDI-156A-7	AmIV	95.5	0.9	2,532	87.5	56	3.05	56.7
CDI-74B-7	AmIV	94.6	0.9	2,502	87.4	48	3.01	56.7
CDI-18B-8	AmIV	94.6	0.0	2,509	88.0	124	2.95	56.9
CDI-148A-8	AmIV	95.5	0.9	2,557	87.3	46	3.04	56.0
CDI-13A-11	AmIV	95.5	0.0	2,670	88.1	66	3.20	56.6
Average^*a*^		93.4	0.2	2,419.7	88.1	68.23	2.87	56.9

a The genome of A. muciniphila Muc^T^ was not included in the averages.

**FIG 1:**
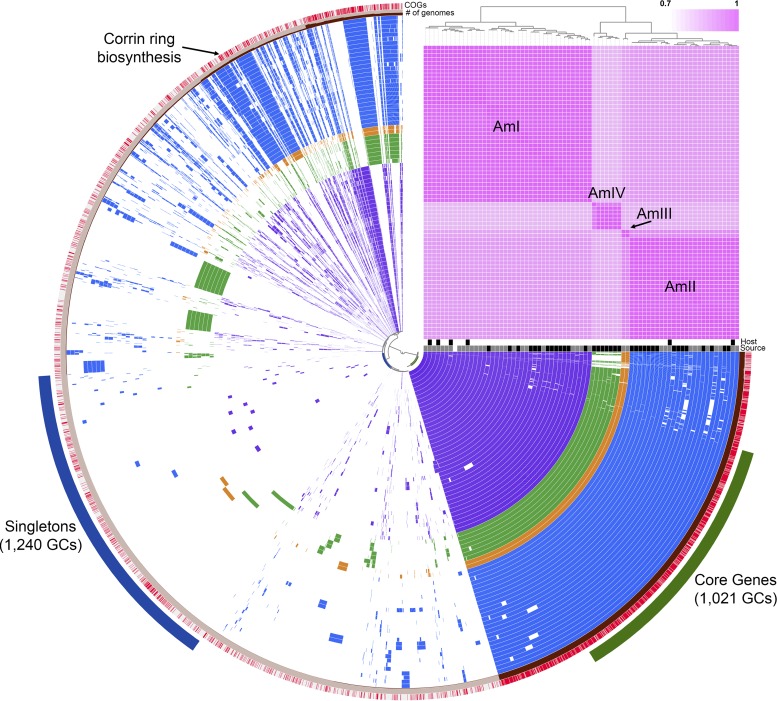
Pangenome of 75 *Akkermansia* genomes generated using anvi’o (17, 18). Each concentric circle represents a bacterial genome, with purple circles belonging to the AmI phylogroup, blue to AmII, orange to AmIII, and green to AmIV. Blank areas in each circle indicate the absence of a particular GC in that genome. A total of 6,557 GCs were observed across all genomes. Genomes are ordered by ANI, as depicted by the pink heatmap in the upper right. Host organisms are indicated below the heatmap, in white (human) or black (mouse) boxes. Similarly, genome sources are indicated in white (12), gray (14), and black (this work) boxes. The outermost ring is colored according to the presence (red) or absence (gray) of functional COG annotations. The next ring indicates the number of genomes in which that particular GC was observed. Singleton (blue) and core (green) genes are indicated outside the concentric circles. Corrin ring biosynthesis genes are indicated in the AmII (blue) and AmIII (orange) genomes.

To explore the genomic diversity of human-associated *Akkermansia* strains, we performed a pangenomic analysis using tools in anvi’o (17, 18). These analyses included the closed genome of the type strain (12) and 33 other human-associated and 6 mouse-associated *Akkermansia* genomes (14). Previously, these 40 *Akkermansia* genomes were used to define three species-level phylogroups, AmI, AmII, and AmIII (14). Merging our 35 MAGs with these 40 other genomes, we were able to regenerate the three original phylogroups and also observed a fourth phylogroup (AmIV), based on average nucleotide identity (ANI) values calculated using PyANI (19) (Fig. 1). Additional phylogenetic analyses of single-copy genes (20) also revealed at least four *Akkermansia* phylogroups (see Fig. S1 in the supplemental material). Phylogroup AmI, which includes the type strain A. muciniphila Muc^T^, contained the largest number of genomes (*n* = 40), followed by AmII (*n* = 26), AmIV (*n* = 7), and AmIII (*n* = 2). Phylogroup AmIII was not observed in any of our 35 MAGs. Interestingly, both AmI and AmII included isolates obtained from mice. Within each phylogroup, ANI values ranged from 93.94% to 99.98% across >65% of each pair of genomes (Fig. S2). All ANI values for between-phylogroup comparisons were <92%. One genome in AmIV (CDI-148A-8) showed lower similarity (on average, ∼94%) to other genomes within this phylogroup, possibly indicating further species-level diversity among human-associated *Akkermansia* strains. Across all phylogroups, we identified 6,557 gene clusters (GCs), with 1,021 being found in all 75 genomes and 1,240 being found in only 1 genome (Fig. 1). Functional genes within the core included the cytochrome *bd* genes (Amuc_1694 and Amuc_1695) (21) and type IV pilus genes (Amuc_1098 to Amuc_1102) (22–24) previously characterized from A. muciniphila Muc^T^.

**FIG 2:**
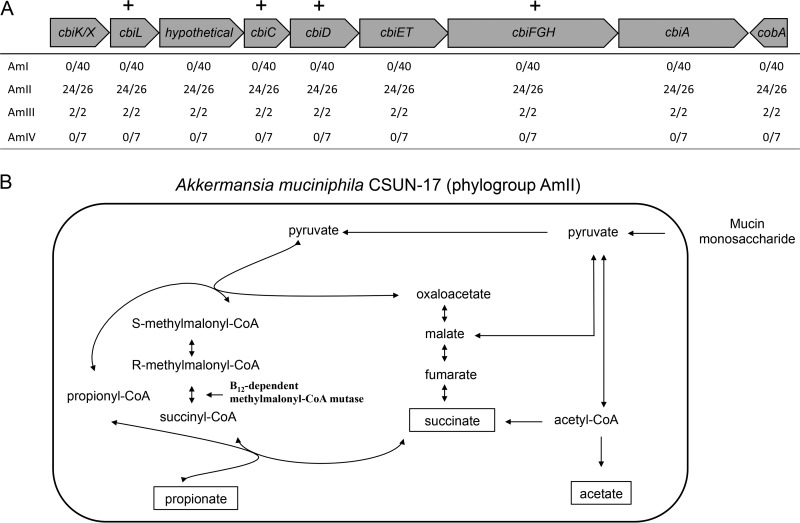
Corrin ring biosynthesis gene cluster from isolate A. muciniphila CSUN-17 (phylogroup AmII). (A) Presence of genes in the corrin ring biosynthesis gene cluster in phylogroups AmI, AmII, AmIII, and AmIV. Plus signs above the table indicate the presence of a gene in A. muciniphila CSUN phylogroup AmII isolates, determined using a PCR screen of A. muciniphila CSUN isolates. (B) Proposed strategy of propionate production in A. muciniphila CSUN-17 (phylogroup AmII), involving *de novo* vitamin B_12_ biosynthesis and leading to activation of methylmalonyl-CoA synthase and conversion of succinate to propionate.

Next, we were interested in identifying functional gene predictions that differed among the phylogroups. Using Clusters of Orthologous Groups (COG) annotations of GCs implemented in anvi’o, we observed 7 GCs putatively involved in the corrin ring stage of cobalamin (vitamin B_12_) biosynthesis within the AmII (24/26 genomes) and AmIII (2/2 genomes) phylogroups (see Data Set S1 in the supplemental material). To investigate these genes in greater detail, we manually inspected the annotations of all 75 genomes using Integrated Microbial Genomes (IMG) (25) and Geneious 7.1.3 (Biomatters, Inc.). With this approach, we confirmed the COG annotations and identified a cluster of 8 genes that appeared to code for the corrin ring biosynthesis proteins in a subset of *Akkermansia* genomes (Fig. 2; also see Data Set S2). Included in this genomic region were genes *cbiK* (or *cbiX*), *cbiL*, *cbiC*, *cbiD*, *cbiET*, *cbiFGH*, and *cbiA*, which encode the enzymes associated with the anaerobic pathway of corrin ring biosynthesis. This cluster also contains a gene whose product is annotated as a hypothetical protein, which shows some similarity to a putative cobalt transporter (26). The content and arrangement of these genes were similar to those of the only other named member of the *Akkermansia* genus, Akkermansia glycaniphila Pyt^T^, which was previously isolated from a python (27). Additionally, all 75 genomes contained most of the genes associated with the upstream (tetrapyrrole precursor biosynthesis, e.g., Amuc_0090, Amuc_0091, Amuc_0417, Amuc_0896, and Amuc_1730) and downstream (nucleotide loop assembly, e.g., Amuc_1678 to Amuc_1683) stages of vitamin B_12_ biosynthesis (28). Genes annotated as a TonB-dependent transporter (e.g., Amuc_1684) and an extracellular solute-binding family 5 protein (e.g., Amuc_1685) that may be involved in vitamin B_12_ import were also identified adjacent to the nucleotide loop assembly genes in all except 1 genome.

A. muciniphila Muc^T^ was previously classified as a cobinamide (Cbi) salvager because it lacks the genes coding for the enzymes to synthesize the corrin ring of vitamin B_12_ but it needs this cofactor for methionine synthesis, nucleotide synthesis, queuosine synthesis, and propionate metabolism (28). Indeed, genes associated with these cellular functions were conserved across all phylogroups (Data Set S1). Interestingly, the vitamin B_12_-independent methionine synthase II gene (*metE*) was present in 25 of 40 AmI genomes but not in any of the other genomes, including that of the type strain Muc^T^. Together, these observations suggest that all *Akkermansia* strains examined here are able to acquire and likely to remodel corrinoids from the environment for use, but some are also able to synthesize this important cofactor *de novo*.

### Cultivation and validation of vitamin B_12_ biosynthesis.

To determine whether specific *Akkermansia* species/strains are indeed able to synthesize vitamin B_12_ *de novo*, we isolated several *Akkermansia* strains from healthy adults and compared their nearly full-length 16S rRNA gene sequences with those reported by Guo et al. (14) in ARB (29), to determine phylogroup affiliation. Across phylogroups AmI, AmII, and AmIII, 16S rRNA gene sequences were all >97% identical but nevertheless clustered into the known phylogroups (Fig. S3). Based on this approach, we identified 8 AmI isolates and 2 AmII isolates in our culture collection. Because our MAGs did not contain any full-length 16S rRNA gene sequences, we could not positively identify AmIV members among the isolates.

Next, using the AmII and AmIII genomes and the genome of A. glycaniphila Pyt^T^, we designed degenerate PCR primers targeting 4 genes, *cbiL*, *cbiC*, *cbiD*, and *cbiFGH*, of the corrin ring biosynthesis GC, which encode a cobalt-factor II C-20-methyltransferase, a cobalt-precorrin-8 methylmutase, a cobalt-precorrin-5B C-1-methyltransferase, and a cobalt-precorrin-4 methyltransferase/precorrin-3B C-17-methyltransferase, respectively (Table 2). These genes were selected because they are predicted to give the best indication of cobamide production, as described by Shelton et al. (28). As expected, only isolates from the AmII phylogroup (CSUN-17 and CSUN-34) and A. glycaniphila Pyt^T^ showed positive amplification, whereas all AmI isolates (including A. muciniphila Muc^T^) failed to show amplification (Table 3). Sequencing and BLAST searching of these PCR amplicons from CSUN-17 against A. glycaniphila strain ERS 1290231 and Desulfovibrio vulgaris strain Hildenborough confirmed the identity of these gene fragments (Table S1), clearly demonstrating the presence of select *cbi* genes in the AmII phylogroup.

**TABLE 2:** PCR primers and bacterial isolates used in this work

Gene or strain	Primer name	Primer (5′ to 3′)^*a*^	Expected amplicon size (bp)	Source	Reference
Gene					
*cbiL*	Precorrin-2 forward	TYTTCAGCATGTCSCGYGAC	358		This work
*cbiL*	Precorrin-2 reverse	GCGGCTRCGGTAGGTYTT	358		This work
*cbiC*	*cbiC* forward	ATCCACACCACGGCRGAC	500		This work
*cbiC*	*cbiC* reverse	GGCGTGCAGGGTRGT	500		This work
*cbiFGH*	*cbiG* forward	GTSAGCAGCGTYTTYG	340		This work
*cbiFGH*	*cbiG* reverse	ATGAGSGCCTGCCKKCCGA	340		This work
*cdiD*	*cbiD* forward	GACCCSGACTGCACSCA	379		This work
*cdiD*	*cbiD* reverse	TAGGCTTCRTGGCTG	379		This work
16S rRNA	8F	AGAGTTTGATCCTGGCTCAG	Variable		79
16S rRNA	515F	GTGCCAGCMGCCGCGGTAA	Variable		79
16S rRNA	806R	GGACTACHVGGGTWTCTAAT	Variable		80
16S rRNA	1492R	TACGGTTACCTTGTTACGA	Variable		81
Strain					
A. muciniphila Muc^T^				Human adult male; ATCC BAA-835	13
A. glycaniphila Pyt^T^				Reticulated python; DSM 100705	27
A. muciniphila CSUN-7				Human adult male	This work
A. muciniphila CSUN-12				Human adult male	This work
A. muciniphila CSUN-17				Human adult male	This work
A. muciniphila CSUN-23				Human adult male	This work
A. muciniphila CSUN-27				Human adult male	This work
A. muciniphila CSUN-28				Human adult male	This work
A. muciniphila CSUN-31				Human adult female	This work
A. muciniphila CSUN-33				Human adult male	This work
A. muciniphila CSUN-34				Human adult male	This work
A. muciniphila CSUN-36				Human adult female	This work

a Y = C or T, S = C or G, B = G, T, or C, and R = G or A.

**TABLE 3:** Presence of select genes associated with corrin ring biosynthesis in CSUN *Akkermansia* isolates, as determined by PCR

Isolate	Phylogroup	Presence or absence^*a*^			
		*cbiL*	*cbiC*	*cbiD*	*cbiFGH*
CSUN-7	AmI	−	−	−	−
CSUN-12	AmI	−	−	−	−
CSUN-17	AmII	+	+	+	+
CSUN-23	AmI	−	−	−	−
CSUN-27	AmI	−	−	−	−
CSUN-28	AmI	−	−	−	−
CSUN-33	AmI	−	−	−	−
CSUN-34	AmII	+	+	+	+
CSUN-31	AmI	−	−	−	−
CSUN-36	AmI	−	−	−	−
A. muciniphila ATCC BAA-835	AmI	−	−	−	−
A. glycaniphila ERS 1290231	NA^*b*^	+	+	+	+

a +, PCR product of the predicted amplicon size.

b NA, not applicable.

**FIG 3:**
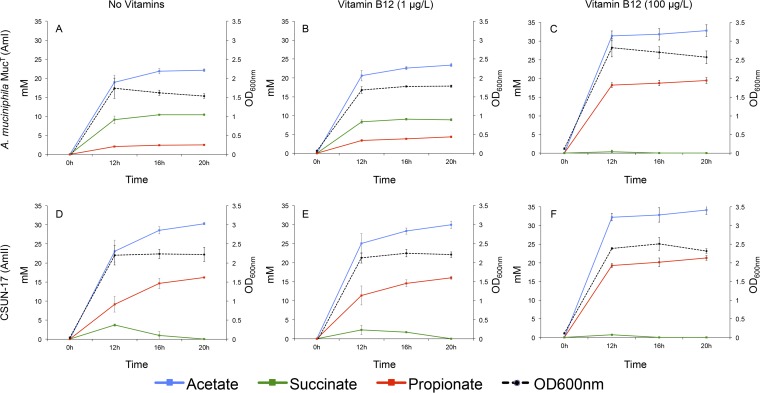
Production of acetate, succinate, and propionate through time by 2 human-associated *Akkermansia* strains grown on purified hog gastric mucin (1%) in the absence of vitamins (A and D), with ATCC MD-VS supplementation (1% [vol/vol] vitamin solution in medium, with vitamin B_12_ at a final concentration of 1 μg/liter medium) (B and E), and with vitamin B_12_ as cyanocobalamin (100 μg/liter) (C and F). All values were averaged from four replicates, and error bars represent the standard deviations. Background levels of organic acids present in the culture medium were subtracted from calculated averages when necessary.

It is known that many fermentative bacteria, including A. muciniphila Muc^T^, use vitamin B_12_ to activate methylmalonyl-coenzyme A (CoA) synthase to convert succinate to propionate (30, 31). Therefore, to demonstrate vitamin B_12_ biosynthesis *in vitro*, we quantified the production of succinate and propionate (and acetate) in the presence and absence of vitamin B_12_ in mucin medium (Fig. 3). Our predictions were that the AmI phylogroup (represented by A. muciniphila Muc^T^) would produce acetate and succinate in the absence of vitamin B_12_ and acetate and propionate when B_12_ was present. For AmII, we predicted that acetate and propionate would be produced regardless of whether the culture medium was supplemented with vitamin B_12_. Results showed that the AmI isolate produced propionate in a vitamin B_12_-concentration-dependent manner (Fig. 3B and C). Also, as expected, the CSUN-17 isolate (AmII) produced significant amounts of acetate and propionate in the absence and presence of exogenous vitamin B_12_, but production was more rapid with supplementation (Fig. 3D to F). These results strongly suggest vitamin B_12_ biosynthesis by strain CSUN-17, which represents the AmII phylogroup.

**FIG 4:**
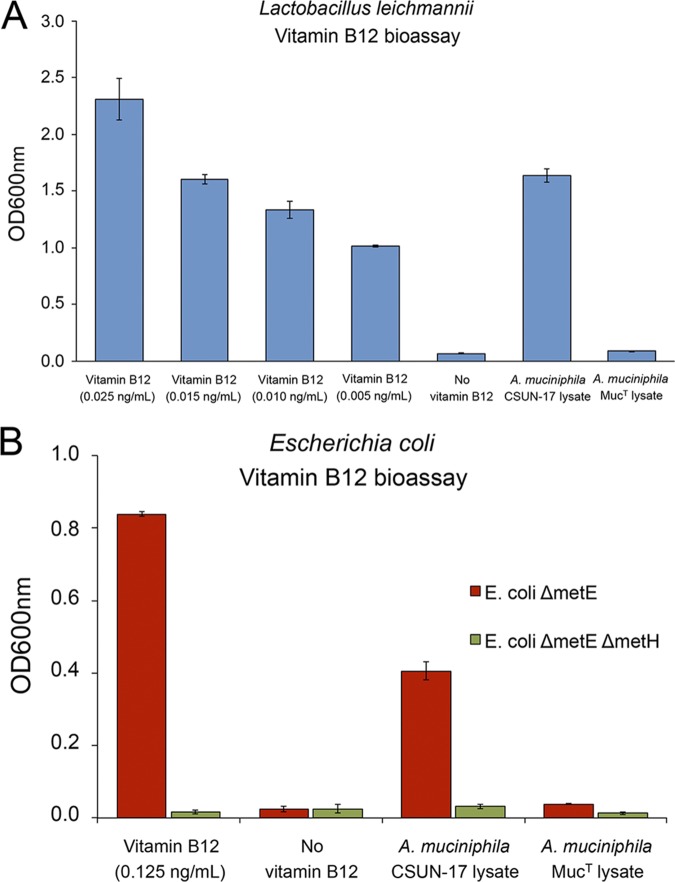
Biosynthesis of vitamin B_12_ by strain A. muciniphila strain CSUN-17, as confirmed by bioassays using L. leichmannii ATCC 7830 (A) and mutant strains of E. coli (B). Growth of L. leichmannii strain ATCC 7830, an E. coli Δ*metE* strain, and an E. coli Δ*metE* Δ*metH* strain was measured in growth medium with and without vitamin B_12_ supplementation and with cell lysates from both A. muciniphila Muc^T^ and A. muciniphila strain CSUN-17. Growth was measured as OD_600_. Values were averaged from three biological replicates, and error bars represent the standard deviations.

Due to the complexity of cobalamin, involving different possible lower-ligand structures, analytical verification of an unknown type of cobalamin can be challenging. Therefore, we used two bioassays to verify *de novo* biosynthesis of vitamin B_12_ by strain CSUN-17. The first bioassay utilized Lactobacillus leichmannii ATCC 7830, which cannot grow without vitamin B_12_ supplementation (32). For the second bioassay, mutant strains of Escherichia coli (E. coli Δ*metE* and Δ*metE* Δ*metH* strains) that require vitamin B_12_ for methionine biosynthesis were utilized (33–36). Results of both bioassays confirmed the biosynthesis of vitamin B_12_ by strain CSUN-17 and not by A. muciniphila Muc^T^, as only CSUN-17 lysates could support the growth of the vitamin B_12_ auxotrophs (Fig. 4).

## DISCUSSION

A. muciniphila is a common gut bacterium that is highly regarded as a beneficial member of the human gut microbiome, with important probiotic potential (10, 37). Various studies have described positive associations between the abundance of *Akkermansia* organisms and intestinal health (3, 4). For example, A. muciniphila affects glucose metabolism and intestinal immunity, and its abundance in the gastrointestinal tract is inversely correlated with diseases, including Crohn’s disease, ulcerative colitis, and acute appendicitis (38–41). Although a number of 16S rRNA gene variants have been observed (12) and dozens of isolates have been obtained (14), human-associated *Akkermansia* strains have largely been considered a single species and the functional potential beyond mucin degradation has gone largely unexplored. Here, we demonstrate that there are significant genomic and physiological differences among the human-associated *Akkermansia* strains. Through comparative genomic analysis, we identified four phylogroups of human-associated *Akkermansia* strains, expanding the known genomic diversity of this lineage. Although all 16S rRNA gene sequences examined here and elsewhere (38) are >97% identical, use of an ANI of 95% across genomes as a species-level delineation (42, 43) would suggest that each phylogroup represents a different species of *Akkermansia*. When we examined gene content, several phylogroup-specific genes that are predicted to code for functional differences among phylogroups were identified, further supporting species delineation. Most notably, we identified a complete set of genes involved in *de novo* biosynthesis of cobalamin, or vitamin B_12_, in two of the four phylogroups. We were able to validate these predictions *in vitro* using novel strains obtained from healthy adults. These findings demonstrate an ecologically important function (44) not previously associated with human-associated *Akkermansia* strains, fundamentally altering our understanding of the diversity and physiology of this lineage. More broadly, these results continue to demonstrate the importance of merging next-generation sequencing approaches with traditional cultivation approaches to elucidate the basic biology of microorganisms of significance.

A recent comparative genomic analysis examining 11,000 bacterial genomes for cobamide production revealed that approximately 37% of bacteria are predicted to synthesize cobamides, although 86% require them for at least one cellular function (28, 45). Additionally, Degnan et al. found that most vitamin B_12_-dependent human gut bacteria lack the ability to synthesize vitamin B_12_ (45). The type strain A. muciniphila Muc^T^ was included in the analysis by Shelton and colleagues (28) and was described as a Cbi salvager able to use exogenous sources of vitamin B_12_. Indeed, based on previous *in vitro* coculture experiments, A. muciniphila Muc^T^ can use at least three types of cobamides, i.e., cyanocobalamin supplied in the culture medium, pseudovitamin B_12_ produced by Eubacterium hallii L2-7 (30), and an unknown form produced by Anaerostipes caccae (46). Presumably, *Akkermansia* strains are able to import these various forms of cobalamin and use them directly or remodel the lower ligand to suit their needs. With our findings, some *Akkermansia* strains can now be considered producers of corrinoids, altering our understanding of how they interact with other members of the human gut microbiome and potentially their human host. However, questions remain regarding the type of cobalamin produced by AmII members and, more generally, the specificity and efficiency of cobamide import and remodeling by all *Akkermansia* strains.

Cobalamin produced by bacteria and archaea in the large intestine is not readily available to the human host, for two main reasons (44). First, the receptors responsible for cobalamin absorption are found in the small intestine, which is not as densely colonized by bacteria as the large intestine in times of health. Second, although bacteria produce many different types of cobalamin, their contribution to the available pool of cobalamin is small because many of the forms produced by bacteria are not recognized by human receptors. Thus, bacteria are thought of more as competitors for dietary cobalamin than as suppliers. However, if a bacterium colonized the small intestine and produced an appropriate form of cobalamin, then the cofactor would possibly be available to the human host. With regard to *Akkermansia* strains, we do not yet know the form of cobalamin produced by AmII members but *Akkermansia*-like organisms have been observed throughout the human gastrointestinal tract, including in the small intestine (reviewed in reference 38). Interestingly, phylogenetic analyses consistently group AmII and AmIII isolates (14) with clones and other sequences previously observed in the small intestine (38). Because our genomic sequence data and isolates were obtained from fecal samples, we could not determine whether the different phylogroups colonize different segments of the gastrointestinal tract, although it is intriguing to speculate.

Although we do not yet know whether humans can directly benefit from vitamin B_12_ produced by *Akkermansia*, there are indirect benefits resulting from the altered metabolites produced when vitamin B_12_ is available. Specifically, the type and quantity of short-chain fatty acids (SCFAs) produced during fermentation influence host health (47–49). For example, propionate is known to help regulate appetite by stimulating the release of peptide YY (PYY) and glucagon-like peptide-1 (GLP-1) by human colonic cells (50). Less is known about the potential benefits of succinate in the human gut, but succinate does improve glucose homeostasis in the mouse cecum via intestinal gluconeogenesis (51). In contrast, succinate has been shown to trigger a type 2 immune inflammatory response, initiated by epithelial tuft cells, in the human small intestine (52). Thus, possessing the ability to synthesize vitamin B_12_ *de novo* would suggest that the AmII and AmIII phylogroups have the potential to consistently produce more propionate than succinate during mucin fermentation and, as a result, influence gut epithelial cell behavior. If the AmII and/or AmIII phylogroups do colonize the small intestine, then the ability to consistently produce propionate over succinate could have significant health implications.

In addition to propionate metabolism, *Akkermansia* strains are predicted to use vitamin B_12_ as a cofactor for methionine biosynthesis using methionine synthase type I (MetH). All genomes possessed the *metH* gene; however, select AmI genomes (25/40 genomes) also contained the vitamin B_12_-independent methionine synthase II (*metE*) gene, suggesting that these select AmI strains can generate methionine in the absence of vitamin B_12_. Given that the AmI phylogroup does not synthesize vitamin B_12_, this would allow production of this essential amino acid when exogenous corrinoids are unavailable. How readily available corrinoids are to *Akkermansia* strains, either from other bacterial producers or from the host diet, is not known, but possessing both variants may be an adaptive strategy for AmI strains.

A. muciniphila is being explored as a commercial probiotic and/or therapeutic agent (41). Recent studies reported large-scale cultivation of A. muciniphila on a defined medium that is safe for human consumption (23) and evaluated the stability and viability of the bacterium in dark chocolate (53). However, our results indicate that there are still gaps in our understanding of the diversity and physiology of human-associated *Verrucomicrobia* strains that need to be explored.

Here, we carried out a pangenomic analysis of 75 *Akkermansia* genomes and identified at least four species-level phylogroups (AmI to AmIV), with differing functional potentials. However, a polyphasic taxonomic characterization that includes robust phenotypic and genomic analyses is needed to verify species designations. Quantification of SCFAs produced by select strains in the presence and absence of vitamin B_12_ supplementation strongly suggested cobalamin biosynthesis by AmII strains. Two bioassays using bacterial strains dependent on vitamin B_12_ for growth confirmed *de novo* biosynthesis of this important cofactor by select *Akkermansia* strains. This work alters our understanding of how *Akkermansia* interacts with its human host and other members of the human gut microbiome in its unique environment. Future work will focus on other genomic similarities and differences identified in our analysis but will also continue to explore vitamin B_12_ production and acquisition using our culture collection. We are also continuing to isolate novel strains from healthy adults, attempting to obtain representatives of each phylogroup that has been observed or others that have yet to be observed.

## MATERIALS AND METHODS

### Metagenomic studies.

**(i) Recruitment and sampling.** Samples used for metagenomic sequencing were obtained from healthy children 2 to 9 years of age, as described elsewhere (54). The participants were informed and provided consent under protocol 1314-223, approved by the institutional review board (IRB) at California State University, Northridge (CSUN). Verbal assent was obtained from each child, and written consent was obtained from one parent/guardian. Data Set S3 in the supplemental material provides unidentifiable demographic information for each child included in this study.

**(ii) DNA extraction, library preparation, and sequencing.** Parents collected fecal samples in the privacy of their homes, using sterile, double-tipped swabs, by swabbing toilet paper (or diapers) after use. Samples were frozen at –20°C within 24 h after collection and transported on blue ice to the laboratory (<30 min in transit), where they were stored at –80°C. This protocol is minimally invasive and has been successfully used in many similar, community-based research projects (55–57).

DNA was extracted from approximately ∼0.1 g of collected samples using the Mo Bio PowerSoil DNA isolation kit (Mo Bio, Carlsbad, CA), following a modified extraction protocol (58). Extracts were then quantified using a Qubit 2.0 fluorometer with high-sensitivity reagents, and 100 ng of DNA from each sample was sheared into 300-bp fragments using a Covaris M220 ultrasonicator (59). The NEBNext Ultra DNA library preparation kit for the Illumina platform (60) was used to prepare dual-indexed metagenomic libraries from the sheared samples. Libraries were confirmed using a Bio-Rad Experion automated electrophoresis system, with Kapa quantitative PCR next-generation sequencing library quantification. Two sequencing runs with the multiplexed libraries were conducted on an Illumina HiSeq 2000 system (2 by 100 bp) at the University of California, Irvine, Genomics High-Throughput Facility.

**(iii) Metagenomic sequence processing.** Raw fastq files from each sample were trimmed using Trimmomatic (61) (with the following parameters: illuminaclip, TruSeq3-PE.fa:2:30:10; leading, 3; trailing, 3; slidingwindow, 4:15; minlen, 36). Trimmed sequences were then screened against the human genome (GRCh38) using DeconSeq (62), in order to remove any potential human DNA sequences. Nonhuman sequences were further cleaned using PRINSEQ (63) (with the following parameters: min_qual_mean, 20; ns_max_n, 3). Remaining sequences without a matepair were removed, and paired sequences were assembled using the default parameters for metagenomes in SPAdes (15). Resulting contigs of >2 kbp were binned using MetaBAT (16), with default parameters, and the taxonomy and completeness of bins were verified against the *Verrucomicrobia* phylum using the taxonomy workflow of CheckM (64). We determined that bins confidently identified as “k_Bacteria (UID2982)” were *Akkermansia*, and we evaluated the quality of those bins further using MiGA (65). Assembled contigs (>2 kbp) from each child with a high-quality *Akkermansia* bin were submitted to IMG-M, where they were annotated using their workflow (25). Both IMG and Geneious 7.1.3 were used to manually inspect annotations of interest. Vitamin B_12_-associated genes were detected by searching for annotations from Enzyme Commission (EC) numbers, IMG terms, the pfam database, and the COG database (25, 66, 67). The annotations included those used by Shelton et al. (28) and Degnan et al. (45).

**(iv) Pangenome analysis.** To explore the *Akkermansia* pangenome, we combined our 35 MAGs with 40 other publicly available genomes in anvi’o (17, 18). Assembled fasta files were first converted to db files using the anvi-script-FASTA-to-contigs-db command, which uses Prodigal (68) to call open reading frames. Each db file was then annotated against the COG database (69) using the anvi-run-ncbi-cogs command with the use-ncbi-blast flag. After generating the genome storage file with the anvi-gen-genomes-storage command, the anvi-pan-genome command was used with the same parameters (num-threads, 12; minbit, 0.5; mcl-inflation, 10; use-ncbi-blast) as outlined by Delmont and Eren (17, 70, 71). The pangenome was visualized and aesthetics were modified using the anvi-display-pan command. To calculate ANI in anvi’o, the anvi-compute-ani command, which utilizes PyANI (19), was used. To identify functions (i.e., COG annotations) that were differentially distributed among the phylogroups, we used the anvi-get-enriched-functions-per-pan-group command, with phylogroups (AmI to AmIV) as the category. To perform phylogenomic analyses, we used the anvi-get-sequences-for-hmm-hits function, which performs an HMM search of the single-copy genes described by Campbell et al. (20) for each genome, aligns them using MUSCLE (72), and then concatenates them into a single fasta file. The fasta file was then input into FastTree (73) for phylogenetic reconstruction using default parameters, and trees were visualized using FigTree version 1.4.3 (https://github.com/rambaut/figtree).

### Cultivation studies.

**(i) Recruitment and sampling.** Fecal samples used for culturing of *Akkermansia* isolates were obtained from healthy adults using swabs, as described previously (56), under CSUN IRB protocol 1516-146. Written consent was obtained from each subject. Collected samples were refrigerated (4°C) and transferred to culture medium (see below) within 24 h after collection.

**(ii) Enrichment, isolation, genomic DNA extraction, and 16S rRNA gene sequencing.** Anaerobic mucin medium was modified slightly from that described by Derrien et al. (13) and contained 0.4 g/liter KH_2_PO_4_, 0.53 g/liter Na_2_HPO_4_, 0.3 g/liter NH_4_Cl, 0.3 g/liter NaCl, 0.1 g/liter MgCl_2_·6H_2_O, 0.4 g/liter NaHCO_3_, 1 mg/liter resazurin, and 10 ml/liter trace mineral solution, as described by Ferguson and Mah (74). The pH of the medium was adjusted to 6.5. The medium was prepared with boiled Milli-Q water under constant gassing with a gas mixture of N_2_/CO_2_ (80:20 [vol/vol]). The culture medium was later modified to include 1 mM l-threonine and 10 g/liter tryptone (Oxoid), as described previously (31). Broth medium was prepared in serum tubes or bottles, which were sealed with butyl rubber stoppers and aluminum crimp caps prior to being autoclaved at 121°C and 15 lb/in^2^ for 15 min. Prior to inoculation, the medium was reduced with autoclaved 0.05% Na_2_S·9H_2_O and supplemented with 0.5% to 1.0% purified hog gastric mucin (type III; Sigma-Aldrich, St. Louis, MO). Purified mucin was prepared by first autoclaving a 5% or 10% solution prepared in 0.01 M phosphate buffer (stock contained 88.46 g/liter KH_2_PO_4_ and 60.97 g/liter K_2_HPO_4_), performing dialysis using a 12- to 14-kDa membrane (Spectra/Por 4; Spectrum Laboratories, Rancho Dominguez, CA), centrifuging the solution twice for 10 min at 10,000 rpm, and filter sterilizing it through 0.2-μm syringe filters (Whatman GE Healthcare Life Sciences, Chicago, IL) into growth medium. For solid medium, Noble agar (Difco, Detroit, MI) was added and plates were poured in an anaerobic chamber (Bactron IV; Sheldon Manufacturing, Inc., Cornelius, OR) under an atmosphere of N_2_/CO_2_/H_2_ (80:15:5 [vol/vol]). All incubations were performed at 37°C in the Bactron IV anaerobic chamber.

Enrichment cultures targeting mucin-degrading bacteria were initiated by transferring fecal swabs into 5 ml of anaerobic mucin medium in serum tubes and performing 10-fold serial dilutions up to 10^−7^. Cultures were incubated for up to 5 days, monitored daily for changes in turbidity, and inspected using phase-contrast microscopy (Zeiss Axioskop). Positive cultures with oval cells in pairs were further diluted in broth medium and/or transferred to solid medium until purity could be verified microscopically and by sequencing of the 16S rRNA gene. For sequencing, genomic DNA was isolated using the Mo Bio Ultraclean microbial DNA isolation kit, following the manufacturer’s instructions. Briefly, 1.8 ml of overnight bacterial culture was centrifuged at 10,000 × *g* for 30 s, the pellet was resuspended in 300 μl of microbead solution (Mo Bio), and the DNA was subsequently isolated following the manufacturer’s instructions. For amplification of the 16S rRNA gene via PCR, 2 μl of extracted genomic DNA was added to 25 μl of GoTaq Green Master Mix (Promega, Madison, WI) and 1 μl of 10 μM universal primers 8F and 1492R, using a final PCR mixture volume of 50 μl (Table 2). PCR was conducted with an Eppendorf Mastercycler Pro S 96-well thermocycler, using a program of initial denaturation at 95°C for 3 min, 30 cycles of 95°C for 45 s, 45°C for 1 min (annealing), and 72°C for 1 min, final extension at 72°C for 7 min, and holding at 4°C. PCR mixtures were purified using the QIAquick PCR purification kit (Qiagen). Initial sequencing of the 16S rRNA gene was performed using either the 8F or 1492R primer on an ABI Prism 3730 DNA sequencer (Laragen Sequencing and Genotyping, Culver City, CA). If cultures were pure and yielded positive results for A. muciniphila in a BLAST search, then the nearly full-length 16S rRNA gene was sequenced with additional primers (515F, 806R, and 8F or 1492R) (Table 2). Sequences associated with each isolate were then assembled in Geneious 7.1.3 and imported into ARB (29), as discussed below. General demographic information about donors is provided in Table S2 in the supplemental material.

**(iii) 16S rRNA gene phylogeny.** To determine the phylogroup affiliations of our isolates, 16S rRNA gene sequences of the isolates described by Guo et al. (14) were first extracted from their genomic sequence data and imported into ARB (29). Once in ARB, gene sequences were aligned with the 16S rRNA gene sequence of A. muciniphila Muc^T^, with secondary structure constraints, and manually inspected, and sequences that were <1,000 bp were discarded. Similarly, 16S rRNA gene sequences of our novel isolates were imported and aligned in ARB. A custom alignment mask excluding nucleotide positions found in less than one-half of all isolates was generated, and masked alignments were imported into MEGA7 (75), where phylogenetic reconstruction was generated using the maximum-likelihood approach. Because we knew the affiliation of the isolates described by Guo et al. (14), we were able to place our isolates in this framework based on placement in the 16S rRNA gene tree.

**(iv) Corrin biosynthesis PCR screen of isolates and gene sequencing.** To amplify conserved regions of corrin-biosynthesis-associated genes, degenerate primers were designed (Table 2). Select corrin-biosynthesis-associated homologous sequences were aligned using BioEdit sequence alignment editor version 7.0.5 (http://www.mbio.ncsu.edu/BioEdit/page2.html) (locus tags of sequences used in the alignments are shown in Table S3). All gene sequences were obtained from JGI IMG/ER. Conserved regions were found using the accessory application ClustalW multiple alignment tool in BioEdit (76). For amplification of the corrin biosynthesis genes *cbiL*, *cbiC*, *cbiD*, and *cbiFGH*, 1 μl of genomic DNA was added to 12.5 μl of GoTaq Green Master Mix (Promega) and 1 μl of 10 μM each primer, using a final PCR mixture volume of 25 μl. PCR conditions were optimized, and a PCR screen of isolates was carried out in duplicate, using a PCR program of initial denaturation at 95°C for 2 min, 25 to 35 cycles of 95°C for 45 s, 52°C to 62°C for 30 s to 1 min (annealing), and 72°C for 45 s, final extension at 72°C for 5 min, and holding at 4°C. PCR amplicons were separated and visualized using a 1% agarose gel. PCR products were purified using the QIAquick PCR purification kit (Qiagen). For amplification of *cbiFGH*, the amplicon was excised from the gel and gel purified using the PureLink quick gel extraction and PCR purification combo kit (Invitrogen). The amplicons were sequenced as described above. BioEdit version 7.0.5 was used to analyze the sequences. Sequences of PCR amplicons from CSUN-17 were checked by BLASTx using the IMG and the NCBI database, to examine similarity to vitamin B_12_-associated genes from the genomes of A. glycaniphila strain ERS 1290231 and Desulfovibrio vulgaris strain Hildenborough (Table S1).

**(v) Quantification of SCFAs via HPLC.** To quantify production of SCFAs with and without vitamin supplementation, A. muciniphila Muc^T^ (AmI) and CSUN-17 (AmII) were grown in anaerobic mucin medium supplemented with 1 mM l-threonine, 10 g/liter tryptone (Oxoid), 1% purified mucin, and vitamin supplementation, depending on treatment conditions. For vitamin supplementation, we first performed the experiment using the ATCC MD-VS supplement at the recommended concentration (10 ml/liter). Because the concentration of vitamin B_12_ in the formulation is 100-fold less than those reported by Belzer et al. (30), we subsequently performed a second experiment with pure vitamin B_12_ (Sigma-Aldrich), using a final concentration of 100 ng/ml. For all experiments, overnight cultures were transferred three times in the appropriate medium, with the final transfer being used to inoculate 25 ml of medium at 5% in quadruplicate for each isolate and treatment. The optical density at 600 nm (OD_600_) (determined with an Eppendorf BioPhotometer Plus) was recorded at inoculation and at 12, 16, and 20 h. An additional 1.25 ml of culture was removed at each time point and centrifuged at 15,000 × *g* for 10 min, and the cell-free supernatant was filtered through a 13-mm, 0.2-μm, Spartan high-performance liquid chromatography (HPLC) syringe filter. Samples were stored at –20°C until HPLC analysis.

HPLC was performed using a Waters Breeze 2 system (Waters Corp., Milford, MA) equipped with a refractive index detector (model 2414). An Aminex HPX-87H column (Bio-Rad Laboratories) was used to measure the production of SCFAs. Sulfuric acid (5 mM) was used as the mobile phase, at a flow rate of 0.6 ml/min. Peak areas and retention times were compared against known standards. Samples were also compared against a medium-only control, to determine background levels of acetate, propionate, and succinate present in the starting medium before growth. Approximately 3 mM propionate was detected in the culture medium and subtracted from all respective measurements.

**(vi) Vitamin B_12_ bioassays.** To confirm vitamin B_12_ production by *Akkermansia* phylogroup AmII, we used two bioassays involving bacterial strains that depend on vitamin B_12_ for growth. For the first bioassay, the classic approach of Hoff-Jørgensen (32), using Lactobacillus leichmannii ATCC 7830 (formerly Lactobacillus delbrueckii subsp. *lactis* ATCC 7830), was employed. Briefly, L. leichmannii was cultured overnight in MRS broth (BD Difco) and incubated at 37°C under an atmosphere with 5% CO_2_. To prepare L. leichmannii for the assay, 0.5 ml of the overnight culture was removed and centrifuged at 15,000 × *g* for 3 min, followed by three washes with sterile Milli-Q water. Washed cells were then incubated at 4°C for 45 min before being inoculated at 0.1% (vol/vol) into 10 ml of sterile vitamin B_12_ assay medium (BD Difco) with standard concentrations (0 to 0.25 ng/ml) of cyanocobalamin (Sigma-Aldrich) or 10 μl of cell extracts of A. muciniphila Muc^T^ (AmI) or CSUN-17 (AmII) (see below). Standard tubes and assay tubes were incubated for 18 to 24 h at 37°C under an atmosphere of 5% CO_2_, and growth was measured as the OD_600_. All experiments were conducted in triplicate and repeated at least twice.

To confirm vitamin B_12_ production by *Akkermansia* strain CSUN-17, a vitamin B_12_-dependent E. coli bioassay was performed using E. coli Δ*metE* and Δ*metE* Δ*metH* mutant strains (33). E. coli MetE is a cobalamin-independent homocysteine transmethylase (34), and E. coli MetH is a cobalamin-dependent methionine synthase (35, 36). The E. coli Δ*metE* strain requires either methionine or vitamin B_12_ supplementation for growth. The E. coli Δ*metE* Δ*metH* strain requires methionine supplementation for growth and was used as an additional control to demonstrate that methionine was not present in the *Akkermansia* extracts at levels that would support E. coli Δ*metE* strain growth. The E. coli Δ*metE* and Δ*metE* Δ*metH* strains were inoculated from LB agar plates into M9 minimal medium supplemented with methionine (1 mg/ml) and were grown for 24 h, to saturation. E. coli cultures were subsequently inoculated at 1% (vol/vol) into fresh M9 minimal medium with methionine (1 mg/ml) and were grown for 24 h. Cell pellets were then washed three times in M9 minimal medium without methionine supplementation before being inoculated to a starting OD_600_ of 0.01 in M9 minimal medium without methionine and being incubated for 24 h at 37°C. E. coli growth was determined by measuring the OD_600_. E. coli mutant strains were examined for growth under five different conditions, i.e., no vitamin B_12_ supplementation, vitamin B_12_ supplementation (0.125 ng/ml; Sigma-Aldrich), 7 μl A. muciniphila Muc^T^ extract supplementation, 7 μl A. muciniphila strain CSUN-17 extract supplementation, and no bacteria (mucin medium control). *Akkermansia* extracts were prepared for E. coli bioassays as described below. Assays were carried out in 2-ml volumes in 15-ml Corning polypropylene tubes. Assays were carried out in biological triplicates, and the experiment was replicated. Negative controls without E. coli were included.

Cell extracts were prepared for both A. muciniphila Muc^T^ (AmI) and CSUN-17 (AmII) by first growing each strain for 18 to 24 h at 37°C in 50 ml of 1% mucin medium, as described above. Extracts were then obtained from each culture by following the protocol described by Kumudha and Sarada (78), with slight modifications. Briefly, cells were pelleted by centrifugation at 10,000 × *g* for 10 min, and the supernatant was discarded. Cells were resuspended in 50 ml of Milli-Q water and autoclaved at 121°C for 10 min. Once cooled, cell extracts were centrifuged again (1,000 × *g* for 10 min) and adjusted to pH 6.0 with HCl, and each supernatant was filtered through a 25-mm, 0.2-μm, polyethersulfone (PES) membrane Whatman syringe filter (GE Healthcare). Extracts were prepared fresh for each bioassay.

For both bioassays, all glassware was baked at 250°C for 2 h to remove organic residues, and standard solutions of cyanocobalamin (product no. V2876; Sigma-Aldrich) were prepared using sterile Milli-Q water. After preparation and filter sterilization through 25-mm, 0.2-μm, PES membrane filters, all standards were kept in the dark and stored at 4°C.

### Data availability.

Genomic sequence data from Guo et al. (14) are available at GenBank under BioProject no. PRJNA331216. Our quality-filtered metagenomic sequence data are available at GenBank under BioProject no. PRJNA525290. Additionally, assembled contigs of >2 kbp from children with an *Akkermansia* bin are available in IMG under GOLD study identification no. Gs0133482. It is important to note that contigs available in IMG include not only *Akkermansia* contigs but all contigs from each child. Data Set S4 has a list of the *Akkermansia* contigs in IMG that were included in our analysis. Nearly full-length 16S rRNA gene sequences of our isolates are available in GenBank under accession no. MK577303 to MK577312. Corrin gene sequences of isolate CSUN-17 are available in GenBank under accession no. MK585566 to MK585569.

## Supplementary Material

Supplemental file 1

Supplemental file 2

Supplemental file 3

Supplemental file 4

Supplemental file 5
