# Supplementary material for: Comparative Genomics Guides Elucidation of Vitamin B12 Biosynthesis in Novel Human-Associated Akkermansia Strains
Source: Appl Environ Microbiol. 2020 Jan 21;86(3):e02117-19. doi: 10.1128/AEM.02117-19 (PMC6974653; doi:10.1128/AEM.02117-19)
Supplement: Supplemental file 1 [file zam003209585s1.pdf]

Supplemental Figure Captions

Supplemental Figure 1. Phylogenetic tree of single copy genes from 75 *Akkermansia* genomes illustrating the four phylogroups. The 'GP' and 'BS' isolates are from Guo et al [1] while the 'CDI' genomes are from this current work. See methods for details on tree construction.

1. Guo X, Li S, Zhang J, Wu F, Li X, Wu D, Zhang M, Ou Z, Jie Z, Yan Q et al. (2017) Genome sequencing of 39 Akkermansia muciniphila isolates reveals its population structure, genomic and functional diverisity, and global distribution in mammalian gut microbiotas. BMC genomics. 18(1):800.

Supplemental Figure 2. Relationship between shared gene content and average nucleotide identity (ANI) within and between Akkermansia phylogroups. Each point represents comparisons between each pair of genomes.

Supplemental Figure 3. Maximum likelihood phylogenetic tree based on 16S rRNA gene sequence comparisons of 47 Akkermansia species illustrating three phylogroups (AmI, AmII, and AmIII). Circles indicate isolate obtained from this work, while the 'GP' and 'BS' isolates are from Guo et al [1]. The tree is rooted with *Rubritalea marina* DSM 17716, another member of the Verrucomicrobia phylum. The evolutionary history was inferred by using the Maximum Likelihood method based on the Tamura-Nei model [2] only considering unambiguously aligned nucleotide positions (n=1305). The tree with the highest log likelihood (-2692.73) is shown. Initial tree(s) for the heuristic search were obtained automatically by applying Neighbor-Join and BioNJ algorithms to a matrix of pairwise distances estimated using the Maximum Composite Likelihood (MCL) approach, and then selecting the topology with superior log likelihood value.

The tree is drawn to scale, with branch lengths measured in the number of substitutions per site. Alignments Evolutionary analyses were conducted in MEGA7 [3].

1. Guo X, Li S, Zhang J, Wu F, Li X, Wu D, Zhang M, Ou Z, Jie Z, Yan Q et al. (2017) Genome sequencing of 39 Akkermansia muciniphila isolates reveals its population structure, genomic and functional diversity, and global distribution in mammalian gut microbiotas. BMC genomics. 18(1):800.

2. Tamura K. and Nei M. (1993). Estimation of the number of nucleotide substitutions in the control region of mitochondrial DNA in humans and chimpanzees. Molecular Biology and Evolution 10:512-526.

3. Kumar S., Stecher G., and Tamura K. (2016). MEGA7: Molecular Evolutionary Genetics Analysis version 7.0 for bigger datasets. Molecular Biology and Evolution 33:1870-1874.

Supplemental Figure 1

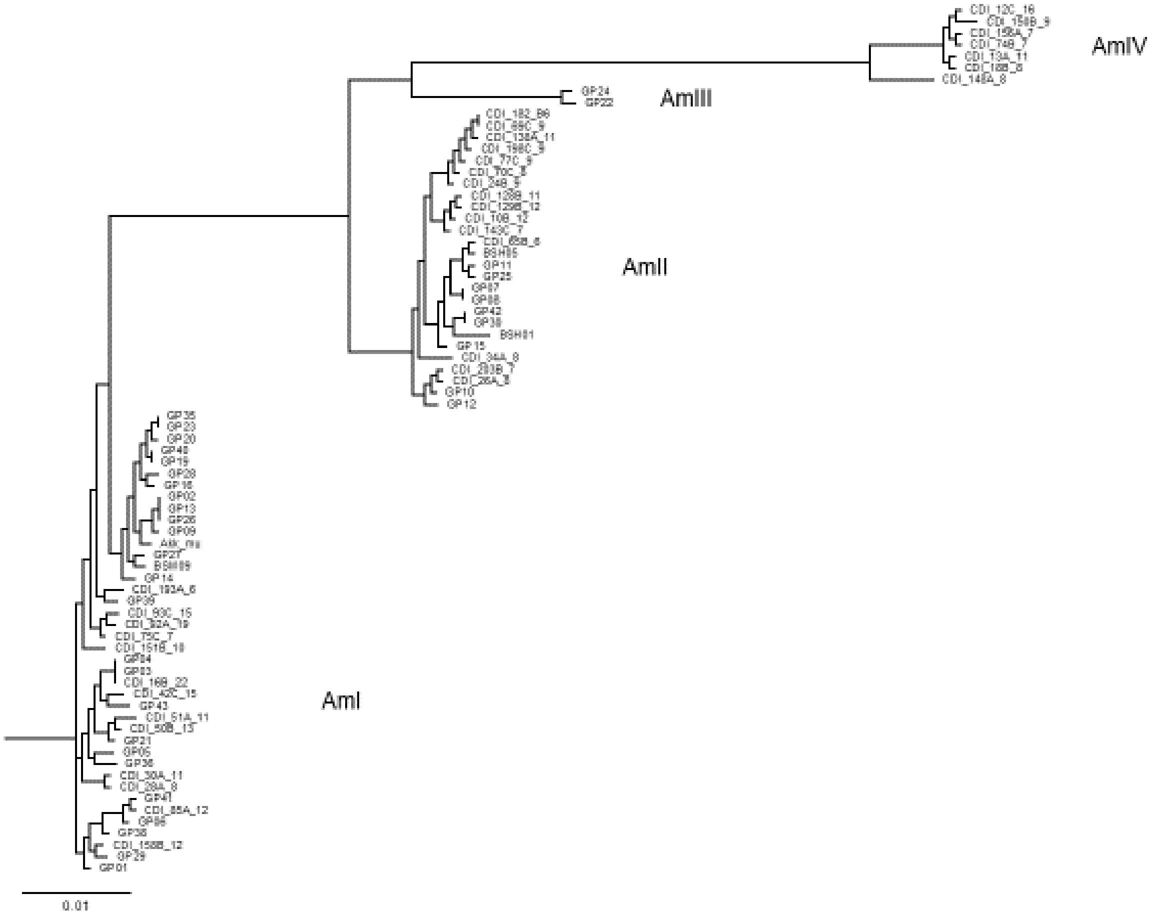

Supplemental Figure 2

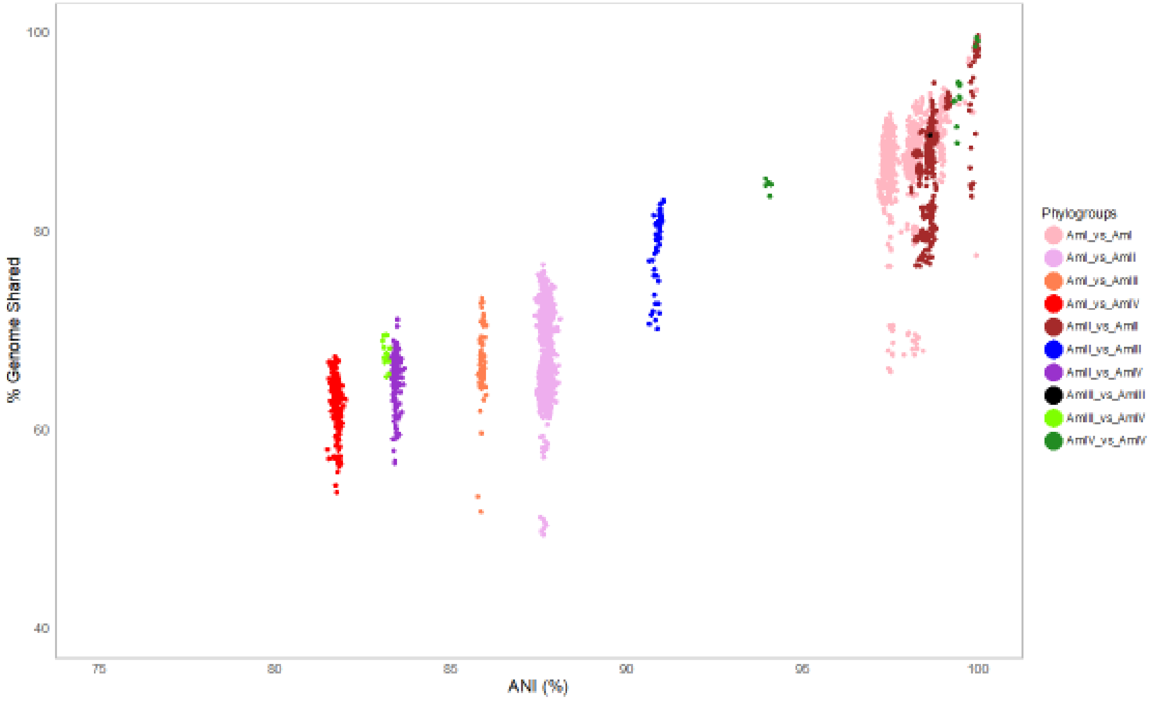

Supplemental Figure 3

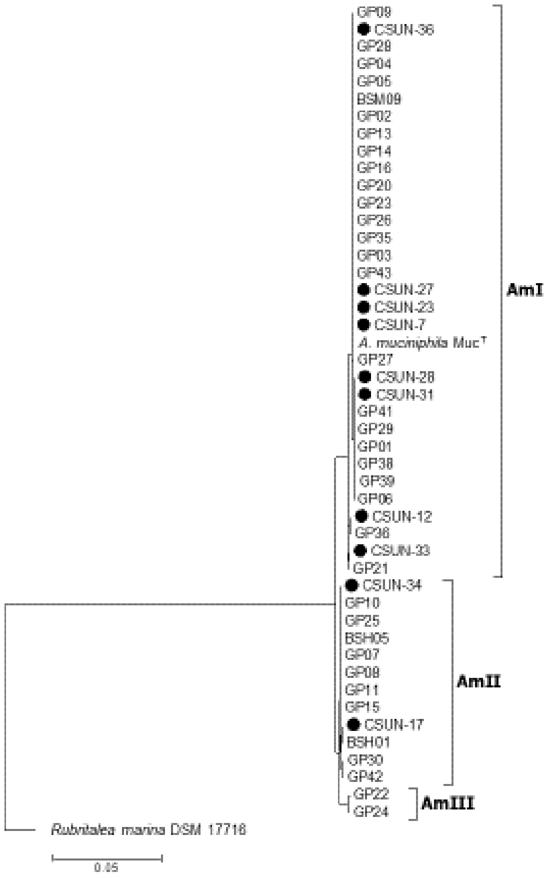

Supplemental Table 1. Corrin ring biosynthesis sequenced amplicons from CSUN-17 homology to *A. glycaniphila* and *Desulfovibrio vulgaris*.

**Gene name**	***A. glycaniphila* gene name**[a]	**Percent identity to *A. glycaniphila***[b]	***Desulfovibrio vulgaris* gene name**[a]	**Percent identity to *Desulfovibrio vulgaris***[b]
cbiL	precorrin-2 C20-methyltransferase /cobalt-factor II C20-methyltransferase	65%	precorrin-2 C20-methyltransferase	48%
cbiC	precorrin-8X methylmutase	80%	precorrin-8X methylmutase	49%
cbiD	cobalt-precorrin-5B (C1)-methyltransferase	70%	cobalamin biosynthesis protein CbiD	46%
cbiFGH	cobalt-precorrin 5A hydrolase/precorrin-3B C17-methyltransferase	56%	precorrin-4 C11-methyltransferase	32%

[a]IMG/ER gene names using BLASTx against *A. glycaniphila* ERS 1290231 and *Desulfovibrio vulgaris* (Hildenborough)

[b]Percent identity of CSUN-17 sequence using BLASTx against *A. glycaniphila* ERS 1290231 and *Desulfovibrio vulgaris* (Hildenborough)

Supplemental Table 2. Demographic information of individuals who provided fecal samples from which strains of *Akkermansia* were isolated.

**Isolate**	**Phylogroup**	**Age**	**Gender**	**Ethnicity**	**Diet**
CSUN-7	AmI	63 years	Male	Caucasian	Omnivore
CSUN-12	AmI	22 years	Male	Hispanic	Omnivore
CSUN-17	AmII	32 years	Male	Caucasian	Omnivore
CSUN-23	AmI	27 years	Male	Caucasian	Omnivore
CSUN-27	AmI	28 years	Male	Caucasian	Omnivore
CSUN-28	AmI	33 years	Male	Hispanic	Omnivore
CSUN-31	AmI	23 years	Female	Hispanic	Vegan
CSUN-33	AmI	22 years	Male	Hispanic	Vegetarian
CSUN-34	AmII	22 years	Male	Hispanic	Omnivore
CSUN-36	AmI	39 years	Female	Caucasian	Omnivore

Supplemental Table 3: Locus tags in JGI IMG of sequences used for corrin ring biosynthesis degenerate primer design.

Gene	Locus tag
cbiL	Ga0175004_112520
	T370DRAFT_00397
	Ga0256628_10023618
	Ga0257029_1018451
	Ga0257030_10082613
	Ga0257062_100012256
	Ga0257032_118796
	Ga0257040_100005132
	Ga0257041_10019915
	Ga0257042_100005192
	Ga0257043_10000156
	Ga0257044_106873
	Ga0257047_1015355
	Ga0257051_1033411
	Ga0257052_10000756
	Ga0257053_10015516
	Ga0257060_10006251
cbiC	Ga0175004_112518
	DESPIG_00351
	Ga0256628_10023616
	Ga0257029_1018453
	Ga0257030_10082611
	Ga0257062_100012254
	Ga0257032_118794
	Ga0257040_100005134
	Ga0257041_10019913

	Ga0257042_100005190<br>Ga0257043_10000158<br>Ga0257044_106871<br>Ga0257047_1015357<br>Ga0257051_1033413<br>Ga0257052_10000758<br>Ga0257053_10015514<br>Ga0257060_10006249
cbiG	Ga0175004_112515<br>HMPREF0179_00024<br>Ga0256628_10023613<br>Ga0257029_1018456<br>Ga0257030_1008268<br>Ga0257062_100012251<br>Ga0257032_118791<br>Ga0257040_100005137<br>Ga0257041_10019910<br>Ga0257042_100005187<br>Ga0257043_10000161<br>Ga0257044_108973<br>Ga0257047_1015360<br>Ga0257051_1033416<br>Ga0257052_10000761<br>Ga0257053_10015511<br>Ga0257060_10006246
cbiD	Ga0175004_112517<br>HMPREF0178_02483<br>Ga0256628_10023615<br>Ga0257029_1018454

	Ga0257030_10082610<br>Ga0257062_100012253<br>Ga0257032_118793<br>Ga0257040_100005135<br>Ga0257041_10019912<br>Ga0257042_100005189<br>Ga0257043_10000159<br>Ga0257044_108971<br>Ga0257047_1015358<br>Ga0257051_1033414<br>Ga0257052_10000759<br>Ga0257053_10015513<br>Ga0257060_10006248
